# Determination of Genotoxicity Attributed to Diesel Exhaust Particles in Normal Human Embryonic Lung Cell (WI-38) Line

**DOI:** 10.3390/biom11020291

**Published:** 2021-02-16

**Authors:** Joong Won Lee, Hee Jae Lee, Young-Joo Lee, Yong-beom Lim, Woo Jong Sim, Ji-Hye Jang, Hye-Ryeon Heo, Hyun Joung Lim, Ji-Won Jung, Jin Sik Kim

**Affiliations:** 1 Department of Chronic Disease Convergence Research, Division of Allergy and Respiratory Disease Research, Korea National Institute of Health, Chungju 28159, Korea; won3@korea.kr (J.W.L.); heejaewith@gmail.com (H.J.L.); jikong0214@korea.kr (J.-H.J.); hhr52@korea.kr (H.-R.H.); hjlim1121@korea.kr (H.J.L.); 2 Department of Materials science and Engineering, Yonsei University, Seoul 03722, Korea; edelstein77777@gmail.com (Y.-J.L.); yblim@yonsei.ac.kr (Y.-b.L.); 3 GLP Center 1, Korea Conformity Laboratories, Bio Division, Incheon 21999, Korea; kjsim@kcl.re.kr

**Keywords:** fine particulate matter, diesel exhaust particles, genotoxicity, micronucleus assay, comet assay, WI-38, gene set enrichment analysis

## Abstract

Several epidemiological studies concluded that inhalation of diesel exhaust particles (DEP) is associated with an increase in the relative risk of lung cancer. In vitro research evaluating the genetic damage and/or changes in gene expression have been attempted to explain the relationship between DEP exposure and carcinogenicity. However, to date, investigations have been largely confined to studies in immortalized or tumorigenic epithelial cell models. Few studies have investigated damage at the chromosomal level to DEP exposure in normal cell lines. Here, we present the genotoxic effects of DEP in normal cells (embryonic human lung fibroblasts) by conventional genotoxicity testing (micronuclei (MN) and comet assay). We show the differentially expressed genes and enriched pathways in DEP-exposed WI-38 cells using RNA sequencing data. We observed a significant increase in single-strand DNA breaks and the frequency of MN in DEP-exposed cells in a dose-dependent manner. The differentially expressed genes following DEP exposure were significantly enriched in the pathway for responding to xenobiotics and DNA damage. Taken together, these results show that DEP exposure induced DNA damage at the chromosomal level in normal human lung cells and provide information on the expression of genes associated with genotoxic stress.

## 1. Introduction

In recent decades, various studies have been conducted on the effects of particulate matter (PM) on human health. There is epidemiological evidence regarding potential causal relations between inhaled exposures of PM and adverse health outcomes [1,2]. Fine particles (diameters < 2.5 µm (PM_2.5_)) easily penetrate the respiratory tract and bloodstream due to their small size and large surface area at the same mass and increases the risk of cardiovascular and respiratory disease [3,4]. Diesel exhaust particulates (DEP) are considered to be the major source of air pollutants and PM_2.5_. DEP are known as a pulmonary carcinogen on the basis of sufficient evidence showing that exposure is associated with an increased risk for lung cancer [5,6]. In animal and cell studies, inhaled DEP have been shown to deposit with high efficiency in the alveolar region of the lungs and cause local inflammation and oxidative stress that can increase the frequency of genomic alteration in cells [7,8,9,10,11]. These results suggest a genotoxic response to DEP is the underlying mechanism for the onset and progression of lung cancer.

DEP derived from diesel engine combustion processes generally consist of polycyclic aromatic hydrocarbons (PAHs), nitro-PAHs, oxygenated derivatives of PAH (ketones, quinones, and diones), heterocyclic compounds, aldehydes, and aliphatic hydrocarbons, as well as reactive metals such as iron, copper, nickel, zinc, and vanadium [12]. However, DEP components can vary by region, type of fuel and engine, and season [13]. Physicochemical properties of the particles (i.e., size, shape, chemical composition, reactivity) determine their hazard toxicity and the mechanisms by which PM induces adverse effects. The DEP material used in this study was national institute of standards and technology (NIST) standard reference material (SRM) 1650b, which is a well-characterized and stable reference material for mechanistic toxicology research in vitro. It enables a valid comparison of the carcinogenic and genotoxic potential of fine particles. SRM 1650 was mutagenic in *Salmonella typhimurium* and used as positive control in Ames test for vehicle air pollutants [14,15].

Studies have shown NIST 1650b can reliably observe hazard effects of DEP, including reactive oxygen species (ROS) generation and inflammatory response as well as DNA alteration [16,17,18]. Results from cell culture experimental models have shown that NIST 1650b elevated the mRNA level of pro-inflammatory cytokines and DNA strand breaks in the adenocarcinoma human alveolar basal epithelial cell line A549 [16,17]. Pohjola et al. indicated DNA adduct formation in SV40-immortalized human epithelial cells (Beas-2b) after exposure to NIST 1650b [18]. However, it should be stressed that most of the DNA damage effects of DEP, including NIST 1650b, was heavily observed in immortalized or tumorigenic epithelial cell models but minimally observed in human normal cells [19]. Furthermore, none of the team has evaluated the DNA strand breakage and chromosomal alteration following NIST 1650b exposure in human normal fibroblast cells.

Therefore, it is necessary to use human normal fibroblast cells to recapitulate some rudimentary toxicological effects of DEP and make these results more relevant to human physiology [20,21,22]. In this study, we aim to provide a meaningful interpretation of genotoxicity using cytogenetic or gene expression markers induced by DEP in human normal lung cells as a model of pulmonary tissue.

Here, we show that DNA strand breakage and micronucleus induction are induced in normal cells (embryonic human lung fibroblasts, WI-38) after exposure to well-characterized standard DEP (NIST 1650b). We first revealed the genotoxicity of DEP by using the conventional genotoxicity test tool in mammalian WI-38 cells. We show the differentially expressed genes and enriched pathways in DEP-exposed WI-38.

## 2. Materials and Methods

### 2.1. Characterization of the Prepared DEP

DEP (SRM 1650b; National Institute of Standards and Technology, Gaithersburg, MD, USA) were purchased from Sigma-Aldrich and dispersed in the serum-free cell culture medium. The prepared DEP were physically and chemically characterized to determine their impact on the tested biological cells. The DEP samples’ particle sizes were determined by dynamic light scattering (DLS). Briefly, the fluctuation of the scattered light intensity, which is caused by the Brownian motion of the particles in suspension, was measured over time. DLS experiment was performed at 25 °C with ELS-1000ZS (Otsuka Electronics, Osaka, Japan). The polydispersity index (PDI), which indicates the quality concerning the stability and the extent of uniformity and homogeneity of the particle emulsions, was also determined. The surface charge of DEP was measured in a serum-free cell culture medium using the same equipment. pH of the DEP solution was measured with a benchtop pH meter (Fisher Scientific, Waltham, MA, USA) to evaluate the compatibility of the sample to the biological experiments. The study of morphology and elemental analysis of DEP was carried out by scanning electron microscopy (Hitachi SU-70, Tokyo, Japan) equipped with energy-dispersive X-ray (EDX) spectroscopy (Horiba EMAX 3.0, Kyoto, Japan). The DEP sample was deposited on a stub paste of sliver (Electron Microscopy Sciences, Hatfield, PA, USA) and then dehydrated in the air. Finally, Pt was coated on the sample. The SEM observations were carried out at 40,000 magnification with 15 kV voltage. To obtain a relative elemental concentration of the DEP, we performed EDX analysis at 3 spots of the DEP surface.

### 2.2. Cell Culture and Treatment

Normal human embryonic lung cells (WI-38) were obtained from the Korean cell line bank. The cells (1 × 10^5^ cells/mL) were cultured in 24-well or 96-well plates and grown in minimum essential medium (MEM; Sigma-Aldrich, St. Louis, MO, USA) supplemented with 10% fetal bovine serum (FBS; Gibco Life Technologies, Carlsbad, CA, USA) and penicillin/streptomycin (100 U, Gibco Life Technologies) in a humidified 5% CO_2_ atmosphere at 37 °C. The DEP was dispersed in a serum-free cell medium, and cells were incubated with DEP at 6 concentrations (0, 25, 50, 100, 200, or 400 µg/mL) for 24, 48, or 72 h.

### 2.3. Cell Viability Assay and Nitric Oxide/Reactive Oxygen Species Assay

To investigate the cytotoxic effect of the DEP, we measured cell proliferation rate using a CCK-8 assay kit (Dojindo). The assay was performed according to the manufacturer’s instructions. As the water-soluble tetrazolium salt CCK-8 reduced by dehydrogenase activities in cells, the kit measured the number of living cells on the basis of the CCK-8 formazan. The WI-38 cells were plated in 96-well microplates at 2 × 10^4^ cells per well, treated with DEP incubated for 24–72 h at 37 °C in a 5% CO_2_ incubator. Thereafter, 10 µL of the cell proliferation reagent CCK-8 was added to the culture medium and incubation continued for 4 h at 37 °C in the 5% CO_2_ incubator. The samples were then shaken for 1 min on a shaker and absorbance was measured at 420–480 nm using a microplate reader (Spectra Max M2, Molecular Devices, San Jose, CA, USA). Nitric concentration was determined using a commercial assay kit (Intron, Nitric Oxide Plus Detection kit, #21023). Intracellular production of ROS was determined using the cell-permeable probe CM-H2DCFDA (Invitrogen, Waltham, MA, USA). WI-38 cells were pretreated with DEP for 2 h then treated with phosphate buffered saline (PBS) containing 2 µM CM-H2DCFDA for 30 min. The fluorescence intensity was immediately measured using fluorescence microscopy.

### 2.4. In Vitro Comet Assay (Single Cell Gel Electrophoresis Assay)

According to Singh et al.’s [23] protocol, we carried out single-cell gel electrophoresis (Comet) assay. For the first layer, the glass microscope slides were coated with 1% normal melting agarose and fully dried. The harvested human lung embryo fibroblast (WI-38) cells (2 × 10^4^ cells/10 μL) were then mixed with 85 μL of 0.7% low-melting agarose and rapidly spread on the first layer. Finally, 85 μL of 0.7% low melting agarose was spread as the top layer. The prepared slides were then soaked in an alkaline lysis buffer (2.5 M NaCl, 100 mM Na_2_-ethylenediamine tetraacetic acid (EDTA), 10 mM Tris-HCl, 1% Triton X-100, and 10% dimethyl sulfoxide (DMSO); pH 10) for 1 h at 4 °C. Thereafter, slides were washed with distilled water for 10 min and placed in a horizontal electrophoresis chamber, followed by electrophoresis in an alkaline buffer (1 mM Na_2_-EDTA, 300 mM NaOH; pH 13) for 25 min at 20 V and 275 mA. The slides were then washed in a neutralization buffer (0.4 M Tris-HCl; pH 7.4) and immersed in 100% ethanol for 1 h. Finally, slides were stained with 100 μL of SYBR Green solution, and images of 300 randomly selected cells were analyzed from each group using a Comet Assay IV analysis system (Instem-Perceptive Instruments Ltd., Suffolk, Halstead, UK).

### 2.5. In Vitro Micronuclei Aassay

Normal human embryonic lung cells (WI-38) were cultured in a 5% CO_2_ atmosphere at 37 °C. After 24 h of incubation, cultured WI-38 cells were treated with test substances and incubation continued. After 20 h from the start of treatment, 4 μL/mL cytochalasin-B (Sigma-Aldrich) was added, and incubation continued for an additional 28 h. The harvested cultures were then incubated with a 0.075 M KCl solution for 3 min and fixed using a methanol/glacial acetic acid solution (3:1 [*v*/*v*]). This fixation step was repeated twice, and resulting cells were dropped onto clean slides. After being air-dried, cells were stained using a Giemsa solution and then observed under a light microscope (Carl Zeiss, White Plains, NY, USA). The micronuclei (MN) were measured per 2000 binucleated cells. Moreover, 500 cells were scored to evaluate the cytokinesis-block proliferation index (CBPI), which was calculated using the following expression: CBPI = (MI + 2MII + 3(MIII +MIV))/total, where MI through MIV represents the number of cells with 1 to 4 nuclei, respectively, and MIII and MIV are both considered to be in their third cycle [24].

### 2.6. RNA Extraction and RNA-Seq

Total RNA was extracted using Qiazol (Qiagen) according to the manufacturer’s protocol. The quantity and quality of the total RNA were evaluated using the Agilent 2100 bioanalyzer RNA kit (Agilent, Santa Clara, CA, USA). The isolated total RNA was processed for preparing mRNA sequencing library using the Illumina TruSeq Stranded mRNA Sample preparation kit (Illumina, San Diego, CA, USA) according to the manufacturer’s protocol. The quality and size of libraries were assessed using the Agilent 2100 bioanalyzer DNA kit (Agilent, Santa Clara, CA, USA). All libraries were quantified by qPCR using CFX96 Real-Time System (Biorad, Hercules, CA, USA) and sequenced on the NextSeq500 sequencers (Illumina) with paired-end 75 bp plus single 8 bp index read run.

### 2.7. Quantifying Gene Expression, Differentially Expressed Gene Analysis, and Gene Set Enrichment Analysis

Potentially existing sequencing adapters and raw quality bases in the raw reads were trimmed by Skewer ver 0.2.2. The cleaned high-quality reads after trimming the low-quality bases and sequencing adapters were mapped to the reference genome by STAR ver 2.5 software. Since the sequencing libraries were prepared strand-specifically by using Illumina’s strand-specific library preparation kit, the strand-specific library option-library-type = fr-first-strand was applied in the mapping process. To quantify the mapped reads on the reference genome into the gene expression values, we used HTSeq ver 0.9.1 with the strand-specific library option, –s reverse, and with the option for overlapping features, –m intersection-nonempty. Other default options were used. The differentially expressed genes between the two selected biological conditions were analyzed by edgeR ver 3.28.1 R package with the default options used. To compare the expression profiles among the samples, we unsupervised clustered the normalized expression values of the selected few hundred of the differentially expressed genes by in-house R scripts. The scatter plots for the gene expression values and the volcano plots for the expression-fold changes and *p*-values between the 2 selected samples also were drawn by in-house R scripts. Genes were ranked by adjusted *p*-value and the sign of the log fold change and gene set enrichment analysis was performed using gene set enrichment analysis (GSEA) [25] using the hallmark gene sets from MSigDB [26].

### 2.8. Statistics

The statistical analyses were performed using SPSS 12.11, and data were expressed as the mean ± standard error (S.E.). A one-way analysis of variance (ANOVA) was applied to all experiment data, and a value of *p* < 0.05 was considered statistically significance.

## 3. Results

### 3.1. Physical and Chemical Characteristics of DEP

**Figure 1:**
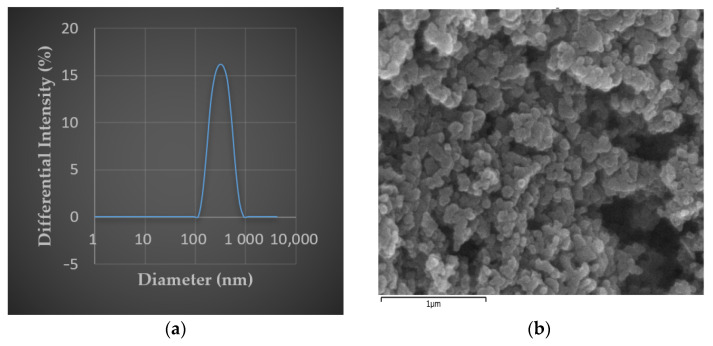
Physical and chemical information on the diesel exhaust particulates (DEP). (**a**) The size distribution of DEP in the cell culture medium, average diameter of 284.2 nm; (**b**) SEM image of DEP (magnification of 40,000×).

DEP (NIST1650b) dispersed in serum-free cell culture medium were analyzed to identify their physiochemical properties. The pH of dispersed DEP was the same as the most mammalian cell culture media in its typical pH 8 formulation [27]. The average size of DEP in the culture medium was 284.2 nm (Table 1, Figure 1a), which was confirmed to be PM_2.5_. The PDI value of DEP samples was 0.164, which indicates a narrow size distribution and homogeneous distribution of DEP [28]. Zeta potential of DEP in the medium was found to be −24.31 mV. This value indicates a weak increase in the agglomerated state of the nanoparticles. Size and zeta potential of DEP measurement were repeated twice each. The SEM image of the DEP is illustrated in Figure 1b. The surface of DEP was seen to be non-uniform. It showed that fine-sized spherical particles appeared to be aggregated. The EDX result in Table 2 displays the average of elemental at three spots of the DEP surface. It indicates the presence of carbon, nickel, copper, bromine, lead, chromium, and sulfur, which are some of the gaseous particulate matter that causes air pollution.

### 3.2. DEP-Induced Cytotoxicity and NO/ROS Production in WI-38 Cells

**Figure 2:**
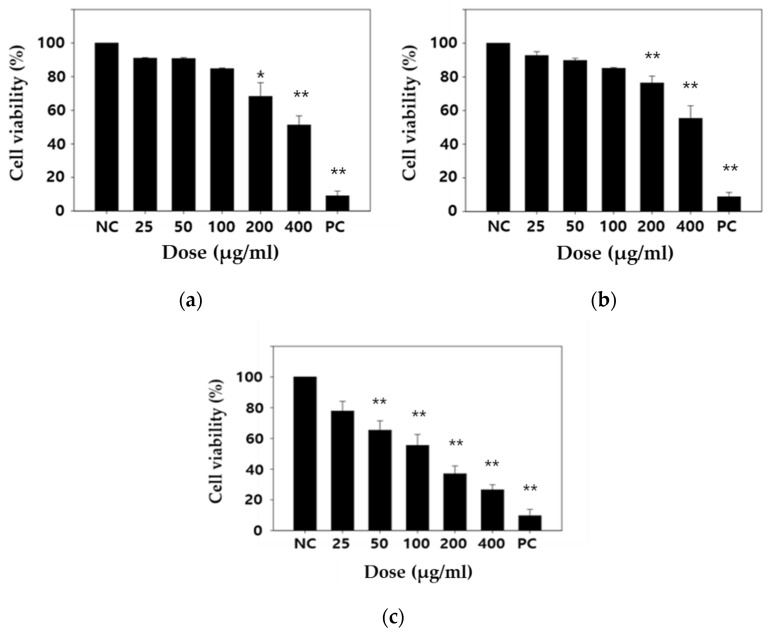
Cytotoxicity measurement data for the human lung embryo fibroblast (WI-38) treated with increasing doses of diesel exhaust particles (DEP). (**a**) The 24 h treatment group; (**b**) the 48 h treatment group; (**c**) the 72 h treatment group. The statistically significant difference is indicated by * *p* < 0.05 and ** *p* < 0.01 when compared with the negative control. Values are means of triplicate measurements. NC: negative control (0.2% DMSO); PC: positive control (1% Triton X-100).

To investigate the cytotoxic effect of the DEP, we measured cell proliferation rate using a CCK-8 assay kit. The cell growth was significantly inhibited at 200–400 μg/mL at 24 and 48 h (Figure 2a,b). The 72 h treatment significantly inhibited cell growth at 50 to 400 μg/mL concentrations (Figure 2c). The DEP significantly and dose-dependently inhibited the WI-38 cells growth level after 72 h of treatment.

**Figure 3:**
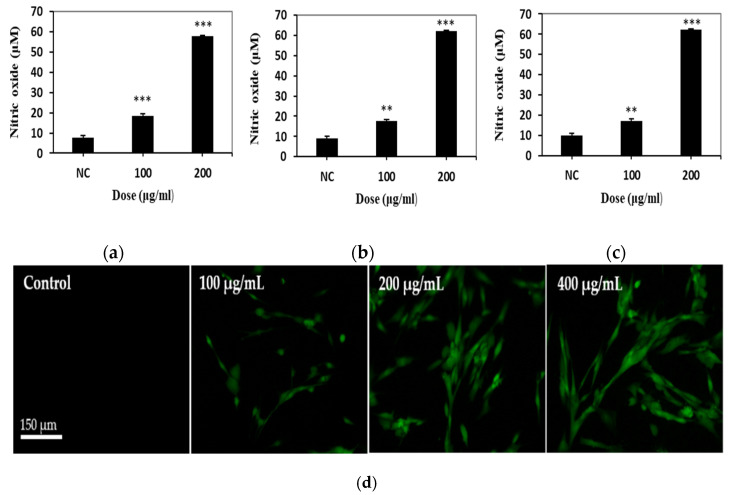
Diesel exhaust particle-induced NO/reactive oxygen species (ROS) production. Changes in nitric oxide levels, after exposure to DEP (0–200 μg/mL) for (**a**) 24 h, (**b**) 48 h, and (**c**) 72 h in WI-38 cells. Statistically significant difference is indicated by ** *p* < 0.01 and *** *p* < 0.001 when compared with the negative control. Values are means of triplicate measurements. NC: negative control. Intracellular ROS production following treatment with DEP (**d**). Incubation with various concentrations of DEP (100–400 μg/mL) increased intracellular ROS production. Scale bar = 150 μm.

The nitric oxide (NO) levels were measured to examine the involvement of oxidative stress in DEP exposure. Exposure to DEP caused significant increases in the NO level in WI-38 cells (Figure 3a–c). These results showed that DEP might induce oxidative stress through the generation of NO in the human lung embryo fibroblast.

To examine whether DEP exposure induces intracellular ROS production, we stained DEP-treated cells using CM-H2DCFDA. After treatment with DEP, the fluorescence intensity indicative of ROS accumulation was significantly higher compared with the unexposed control cells (Figure 3d).

### 3.3. DEP-Induced Genotoxicity in WI-38 Cells

**Figure 4:**
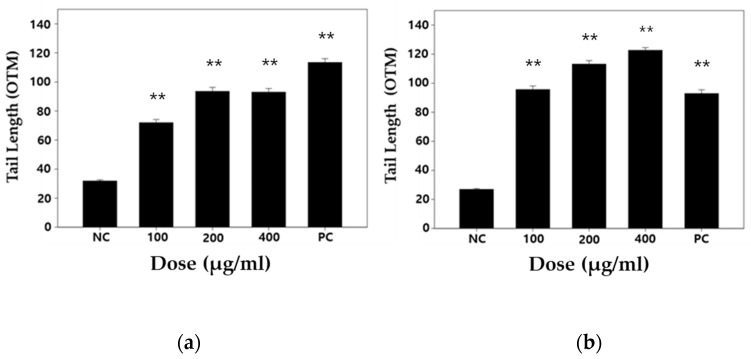
Quantitative assessment of DNA damage in the diesel exhaust particles (DEP) treatment of the human lung embryo fibroblast (WI-38) cells using single-cell gel electrophoresis (comet assay). The slides were stained with SYBR green and analyzed using a fluorescent microscope and image program. (**a**) The 24 h treatment group; (**b**) the 48 h treatment group. A statistically significant difference is indicated by ** *p* < 0.01 when compared with the negative control. Values are means of triplicate measurements. NC: negative control (0.2% DMSO); PC: positive control (0.4 μg/mL mitomycin C).

**Figure 5:**
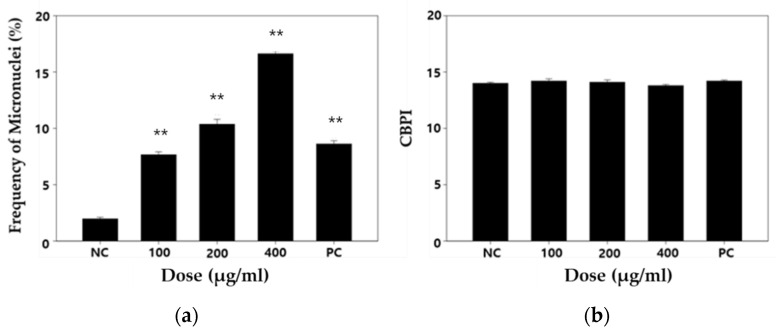
Measurement of (**a**) micronuclei frequencies and (**b**) cytokinesis block proliferation index (CBPI) in the diesel exhaust particles (DEP) treatment of the human lung embryo fibroblast (WI-38) cells. A statistically significant difference is indicated by ** *p* < 0.01 when compared with the negative control. Values are means of triplicate measurements. NC: negative control (0.2% DMSO); PC: positive control (0.4 μg/mL mitomycin C).

The DNA damaging effect produced by DEP treatment was investigated on the basis of single-cell gel electrophoresis (comet assay) and a cytokinesis-block micronucleus (CBMN) assay. Mitomycin C (MMC) was used as a positive control [29]. MMC has been known as a DNA cross-linking agent [30,31]. For DEP-treated WI-38 cells, the olive tail moment (OTM) was higher than negative control cells after 24 and 48 h (Figure 4). As regards the formation of micronuclei (MN), DEP-treated WI-38 cells exhibited many more than the control cells (Figure 5a). It was observed that the micronuclei frequency was dose-dependently increased by DEP treatment. However, DEP could not change the cytokinesis block proliferation index (CBPI), which is a biological index for detecting cytotoxicity or a cell cycle delay (Figure 5b).

### 3.4. DEP Exposure and the Differential Expression Genes in WI-38 Cells

Gene set enrichment analysis (GSEA) was employed to understand the signaling pathways of cellular response to DEP. We performed RNA sequencing to identify genes and pathways involved in DEP-induced DNA damage and xenobiotic response in WI-38 cells. Volcano plots for differentially expressed genes are shown in Figure S1. When compared with vehicle controls, WI-38 cells treated with 200 μg/mL for 24 h displayed 2273 of 19,391 genes with differential expression, 1062 genes had decreased expression levels, and 1211 genes had increased expression levels upon treatment with DEP. GSEA was capable of clearly detecting biologically relevant molecular signaling enrichment in WI-38 cells following exposure to DEP. GSEA of hallmark gene sets representing well-defined biological states showed that 5 gene sets were significantly downregulated and 17 gene sets were upregulated (false discovery rate (FDR) *q*-value < 0.25) (Table 3). Among the significantly downregulated hallmark pathways were several gene sets important for a genotoxic response such as signaling through proliferative pathways (G2/M checkpoint), mitotic spindle assembly, response to ultraviolet (UV) radiation (down), and DNA repair (Table 3). Among the significantly upregulated hallmark pathways were DNA damage/p53 and apoptosis, indicating that DEP exposure drives significant cell death signaling. There was also a statistically significant positive enrichment of the known genes in xenobiotic metabolism and inflammatory response (Table 3). The hallmark gene set for both Kras signaling and mTORc signaling genes was enriched in the DEP-exposed cells when compared to those from unexposed cells (Table 3).

**Figure 6:**
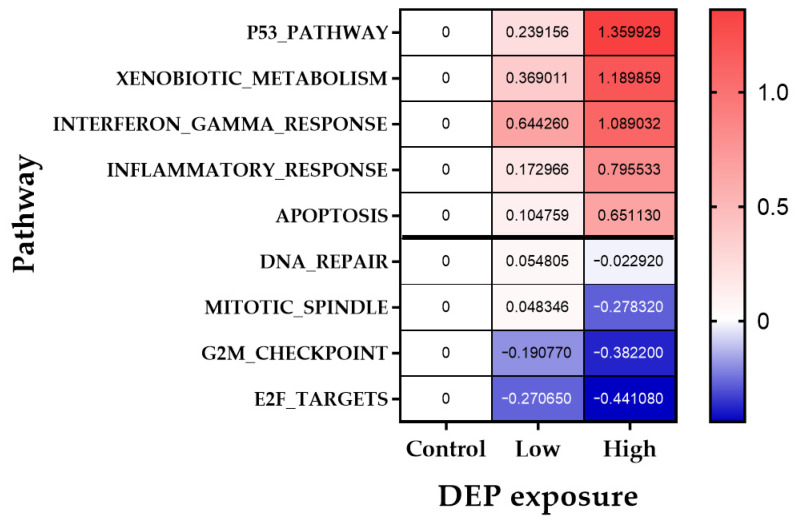
Heatmap of gene set expression of hallmark pathways of interest. Heat map representing the log_2_ fold change values between unexposed control and DEP treatment in the human lung embryo fibroblast (WI-38) cells. Low: 100 μg/mL DEP; high: 200 μg/mL DEP.

We further identified the dose-dependent relationship for the effect of DEP on gene expression data. We further confirmed the dose dependence of DEP exposure effect on the gene expression profile. In WI-38 cells, after 24 h of exposure to 100 and 200 μg/mL of DEP, the expression of gene set of genotoxic stress-related hallmark pathway including p53, xenobiotic metabolism, inflammatory response, DNA repair, and G2-M DNA damage checkpoints was amplified (Figure 6).

## 4. Discussion

The fine particle in this study was standard reference material generated by diesel engine emissions (NIST 1650b). This material consists of several PAHs, nitro-PAHs, elemental carbon, organic compounds, sulfates, nitrates, and trace amounts of metals and other elements [32,33] well known to cause ROS, inflammatory reactions, and DNA damage. NIST SRM 1650b’s characterized mean is 0.18 μm, which makes it feasible to identify the potential health risk of fine range DEP. In this study, we dispersed DEP in MEM cell culture media without serum, and physicochemical properties of aqueous DEP were analyzed by DLS and SEM-EDX. The average size of prepared DEP in media was 0.28 μm, indicating that prepared DEP in the aqueous environment is in the fine particle range, which is small enough for penetrating the lung barrier and enter circulation (Table 1). Prepared DEP in MEM remained approximately stable in the dispersed state. However, the measured zeta potential of DEP in MEM media was −24.31 mV (Table 1), which was less negative than the certain value, it indicates better stability of the colloidal suspension [34]. The shape of the DEP was also analyzed by SEM, which revealed that the particle surface is spherical (Figure 1).

Genotoxic events have been known as a crucial step in the initiation of cancer. In various cell model research projects, DNA damage appeared as an important mechanism of action of DEP-induced pulmonary carcinogenesis [35]. The results obtained from several human cancer cell lines such as A549 (human alveolar carcinoma), THP-1 (human monocyte), HepG2 (human hepatoma), and MCL-5 (human B lymphoblastoid) showed the DEP or polycyclic organic matter derived from DEP induced significant DNA damage [36,37,38,39]. Similar results were observed for the DEP component including NIST 1650a in immortalized human BEAS-2B cells [40,41,42,43]. There is a report that used normal human bronchial epithelial cells (NHBE) for investigating effects of DEP on CYP1A1 level [19]. However, human normal cells have rarely been used for in vitro genotoxicity tests of DEP. Although the genotoxic effects of DEP are relatively well-reported, to validate these findings and yield more biologically relevant data, normal human cells retained most of the characteristics of normal cell growth and differentiation cell should be compared [44].

In the present study, exposure to DEP induced oxidative stress through generation of ROS and NO in WI-38 cells (Figure 3). The imbalance between the production and elimination of ROS causes oxidative stress, which has been associated with aging, carcinogenesis, and Alzheimer’s disease [45,46]. Genotoxicity testing is important to provide adequate hazard identification and risk assessment in terms of the carcinogenesis process [47]. It is well known that fine particle fraction including DEP induces genotoxicity for two main biological processes: ROS production and increased damage in the cell. The former step occurs because DEP consists of redox-active components that can trigger ROS generation upon interaction with a cell [48,49,50]. In the latter case, these reactions lead to interaction with the DNA sequence and chromosome, inducing genomic instability and leading to cell division dysfunction either directly or indirectly inducing microtubule depolymerization and alterations in the spindle structure [51,52,53,54]. As a consequence, structural or numerical aberrations of the genetic material are induced through this process.

We applied two in vitro genotoxic tests to determine whether the DEP induces genetic alteration in normal human lung cells. Comet assay is the most common method for DNA strand breakage detection in individual cells [55]. Moreover, most studies have shown the positive association between DEP exposure and DNA strand breakage using this test. Therefore, first, we performed an alkaline-based comet assay to sensitively measure DEP-induced double- and single-strand breaks, alkali labile sites, DNA cross-linking, and incomplete excision repair sites [23,56]. In this study, we used Comet Assay IV analysis system, which has high sensitivity compared to Comet Analyzer [57]. As a result of the comet assay in WI-38 cells, DEP exposure enhanced the OTM value to a statistically significant level when compared with the control (Figure 4). Up to 24 h of exposure, the intensity of toxic expression 200 μg/mL and 400 μg/mL similarly affected cells. However, at 48 h, 400 μg/mL seemed to be a phenomenon that maintained DNA damage at a higher level than 200 μg/mL. Our comet assay result is in line with the consistent results from several previous studies that have shown DNA damage responses following exposure to DEP [36,37,38,39,42]. The study of DNA alteration at the chromosome level is an essential part of genotoxicity testing because the chromosomal mutation is a critical event in carcinogenesis. Analyses of micronuclei (MN) formation belong to the most commonly used cytogenetic methods to assess a broad-spectrum of DNA damage at the chromosome level including chromosome breakage, chromosome loss, chromosome rearrangement (nucleoplasmic bridges), and cell division inhibition [58]. To further elucidate whether the increased level of DNA strand breaks observed in our comet assay may be related to DNA damage at the chromosome level, we also applied the cytokinesis-block micronucleus assay. MN results from a broken chromosome fragment or an entire chromosome that remains outside the nucleus after mitosis. MN can be formed following direct DNA damage (clastogenic mechanism) or following secondary disturbance of the mitotic apparatus (indirect aneugenic mechanism) [59]. In our MN assay, we showed dose-dependent MN induction of WI-38 cells upon exposure to DEP, indicating that the DEP can induce disruption or breakages of chromosomes in normal lung cells (Figure 5). Collectively, our two in vitro genotoxic tests’ positive results suggest that MN formation after treatment with DEP come from clastogenic effects, although an aneugenic effect was not excluded. To our knowledge, there are few in vitro micronucleus tests for DEP exposure or evaluation of genotoxicity of DEP in normal fibroblast cell lines, and thus this is the first time experimental evidence of MN induction by DEP has been provided [17,60]. Further studies to determine the aneugenic effect of DEP should be performed by alternative cytogenetic methods such as micronucleus assay with fluorescence in situ hybridization [61].

Next, we analyzed the genome-wide expression profiles of normal human lung fibroblast WI-38 cells that had been exposed to DEP. To our knowledge, this is the first study to use an untargeted RNA-sequencing technique to identify differential gene pathways for DEP exposure on human normal fibroblasts. The cytotoxic concentration of DEP (200 ug/mL) for 24 h increased the abundance of differentially expressed downregulated genes in the cell cycle regulation, E2F and G2M checkpoint, and the mitotic spindle assembly pathway (Table 3). In contrast, differentially expressed upregulated genes were involved in the cellular response to cytokines (TNF-α signaling via NF-kB), inflammation, DNA damage, p53, apoptosis, redox sensing signaling (mTORc1), and metabolism of xenobiotics pathway (Table 3). NIST 1650b dose-dependent transcriptomic response was enriched in the cellular response to DNA damage (p53, E2F, G2M, mitotic spindle, DNA repair), xenobiotic metabolism, and inflammation-related pathways (Figure 6). Defects in DNA damage response (DDR) strongly cause various diseases including cancers [62]. Downregulated E2F pathway-related gene reflects limited cell proliferation in response to DNA damage. The E2F transcription factors are key players in cell cycle progression and link the G2M transcription [63]. Cells with a defective G2-M checkpoint, if they enter M-phase before repairing their DNA, facilitate apoptosis [64]. Collectively, we demonstrate that DEP induces defects in DDR mechanisms, including check-point activation and DNA repair, leading to apoptosis in WI-38 cells. Cellular responses to xenobiotic exposure have a critical role in the mechanisms of chemical carcinogenesis [65]. Xenobiotic metabolism acts in detoxifying and eliminating potentially harmful compounds. A zebrafish model study showed that the metabolism of xenobiotics by cytochrome P450 was the critical pathway in PM-induced toxicity [66,67]. The literature review effectively demonstrates that the particulate and organic components of DEP alter the enzyme capacity to metabolizing xenobiotics [68]. In this study, DEP exposure was also significantly enriched in the pathway for responding to xenobiotics in human normal cells. This finding indicates that the xenobiotic metabolic activity may also have a value in predicting response to DEP. Taken together, our GSEA analysis results indicated that DEP might suppress DNA damage repair mechanism, disturb xenobiotic metabolism, and actives p53 and apoptosis pathways in normal human lung cell. However, this finding has the limitation that we did not validate DEG identified using RNA-sequencing. A further mRNA validation study would be necessary to support our findings.

## 5. Conclusions

Our study shows for the first time that exposure to DEP could induce DNA damage at the chromosomal level and induce marked changes in gene expression patterns in human normal lung WI-38 cells. Our data provide comprehensive information on the chromosomal structural and genome-wide transcriptional changes that are induced by well-characterized DEP reference materials in normal human lung cells.

## Figures and Tables

**Table 1:** Characteristics of the prepared fine particles.

Particle	Media	pH	Average Diameter (nm)	PDI	Zeta Potential (mV)
DEP (NIST 1650b)	MEM ^1^	8	284.2	0.164	−24.31

^1^ Minimum essential medium.

**Table 2:** Elemental composition of diesel exhaust particulate matter (PM).

Element	wt %	Element	wt %
C	95.15	Cl	0.18
Ni	1.34	Ca	0.13
Cu	1.32	Mn	0.12
Br	0.87	Fe	0.02
Pb	0.34	As	0.01
Cr	0.32	Si	0.01
S	0.19		

**Table 3:** Gene set enrichment analysis (GSEA) results according to the MSigDB hallmark gene sets.

Hallmark Pathway	Number of Genes in Pathway	NES	Nom *p*-Value	FDR *q*-Value
**Downregulated**				
1. E2F targets	36	2.28	0	0.002
2. G2M checkpoint	43	2.26	0	0.001
3. Mitotic spindle	28	2.23	0.002	0.001
4. UV response dn	32	1.52	0.056	0.077
5. DNA repair	19	1.46	0.075	0.084
**Upregulated**				
1. TNF-α signaling via nfkb	72	−2.5	0	0.004
2. UV response up	33	−2.35	0	0.002
3. p53 pathway	43	−2.3	0	0.001
4. Estrogen response early	38	−2.13	0.002	0.005
5. Xenobiotic metabolism	29	−2.09	0.002	0.005
6. Interferon gamma response	57	−1.96	0.004	0.013
7. Kras signaling dn	33	−1.78	0.014	0.038
8. Inflammatory response	36	−1.71	0.013	0.058
9. Estrogen response late	41	−1.7	0.027	0.053
10. mTORc1 signaling	30	−1.67	0.025	0.059
11. Apoptosis	30	−1.63	0.027	0.067
12. Adipogenesis	17	−1.62	0.043	0.064
13. Hypoxia	44	−1.6	0.021	0.065
14. Unfolded protein response	18	−1.55	0.055	0.082
15. Interferon-alpha response	36	−1.38	0.101	0.17
16. Myogenesis	27	−1.34	0.131	0.195
17. IL-2 stat5 signaling	47	−1.33	0.120	0.194

GSEA of downregulated or upregulated genes in DEP-treated WI-38 cells (200 μg/mL for 24 h) compared to vehicle control. NES = normalized enrichment score. Enrichments were considered significant if false discovery rate (FDR) < 0.25. Nom = nominal.

## Data Availability

Not applicable.

## Supplementary Materials

The following are available online at https://www.mdpi.com/2218-273X/11/2/291/s1, Figure S1: Volcano Plot of differential expressed genes (DEGs), Figure S2: Representative enriched gene set.
