# Supplementary material for: Determination of Genotoxicity Attributed to Diesel Exhaust Particles in Normal Human Embryonic Lung Cell (WI-38) Line
Source: Biomolecules. 2021 Feb 16;11(2):291. doi: 10.3390/biom11020291 (PMC7919825; doi:10.3390/biom11020291)
Supplement: Supplementary file 1 [file biomolecules-11-00291-s001.pdf]

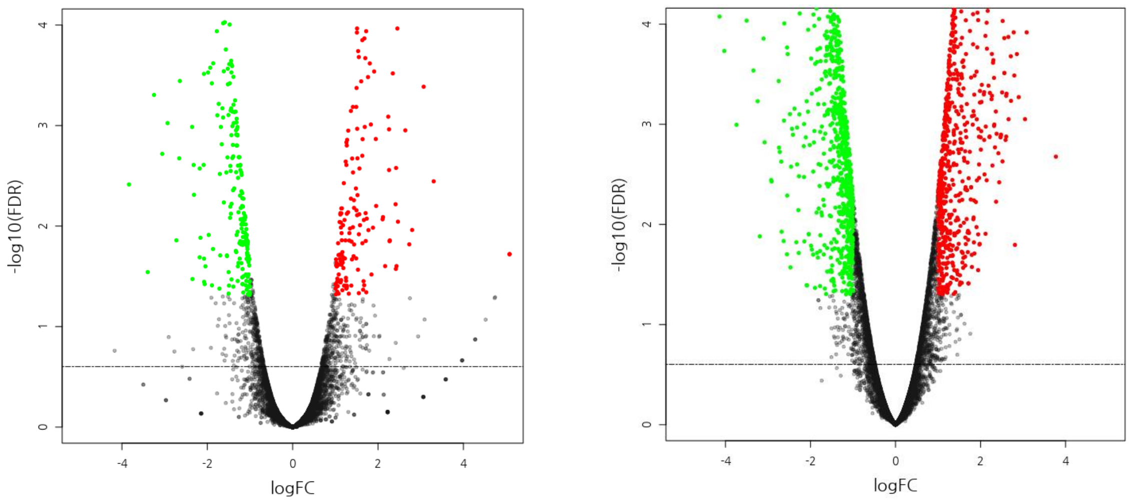

(a) (b)

Supplemental Fig. 1. Volcano Plot of differential expressed genes (DEGs). Volcano Plot showing DEGs between unexposed control and (a) low DEP treatment or (b) high DEP treatment in the human lung embryo fibroblast (WI-38). Upregulated genes are marked in light red; downregulated genes are marked in light green. DEGs were selected with thresholds of false discovery rate (FDR) < 0.25. Low: 100 ug/ml DEP ; High: 200 ug/ml DEP.

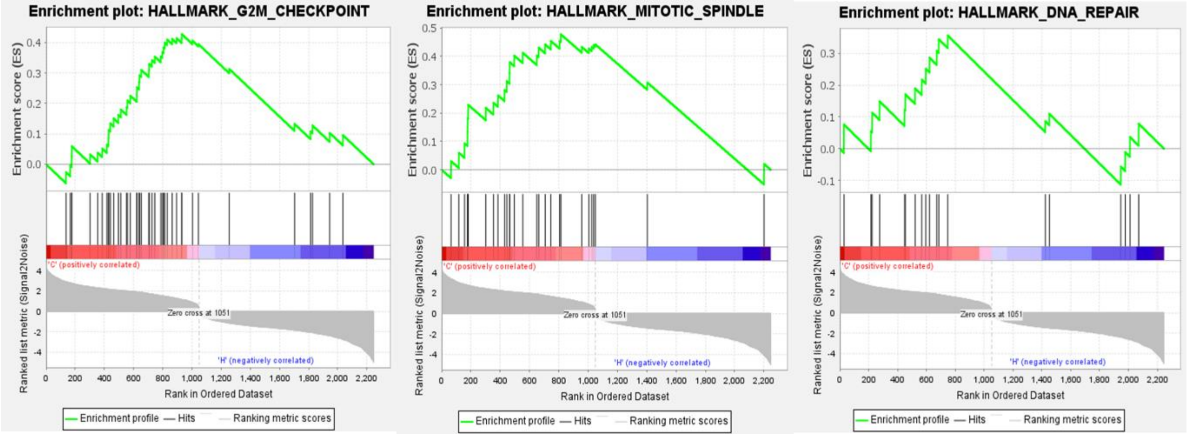

(a)

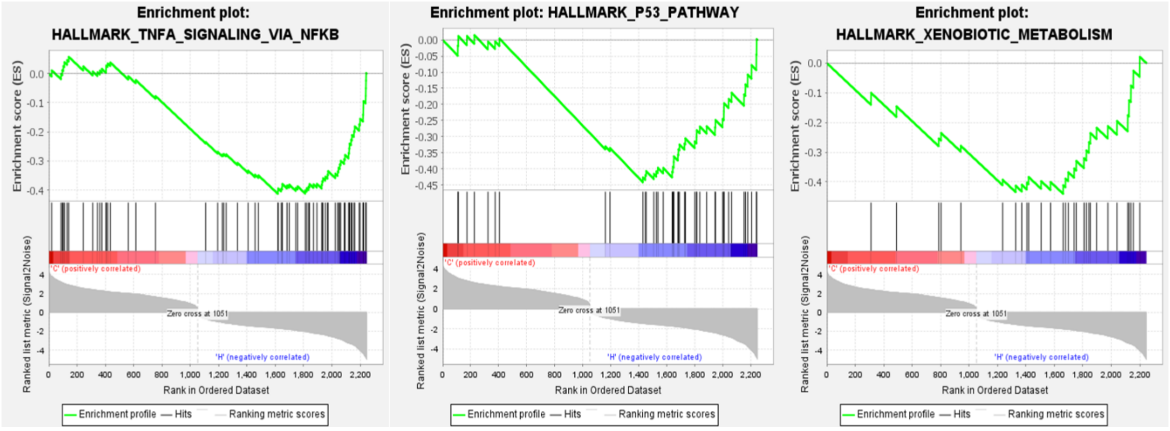

(b)

Supplemental Fig. 2. Representative enriched gene set. Enrichment plots for (a) down-regulated hallmark pathway and (b) up-regulated hallmark pathway.
